# Supplementary material for: Di-Tyrosine Crosslinking and NOX4 Expression as Oxidative Pathological Markers in the Lungs of Patients with Idiopathic Pulmonary Fibrosis
Source: Antioxidants (Basel). 2021 Nov 18;10(11):1833. doi: 10.3390/antiox10111833 (PMC8615037; doi:10.3390/antiox10111833)
Supplement: Supplementary file 1 [file antioxidants-10-01833-s001.zip › antioxidants-1446701-supplementary.pdf]

Supplementary Materials:

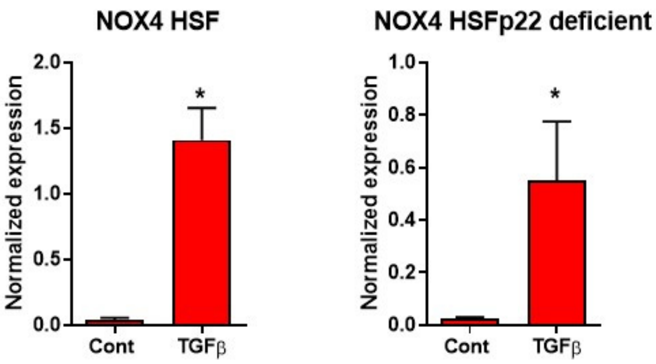

Figure S1. TGFβ1-induced upregulation of the expression of NOX4 in human skin fibroblasts from healthy donor (HSF) and CYBA-deficient patient (HSFp22 deficient). N = 4, * $p < 0.05$ using Mann Whitney nonparametric test.

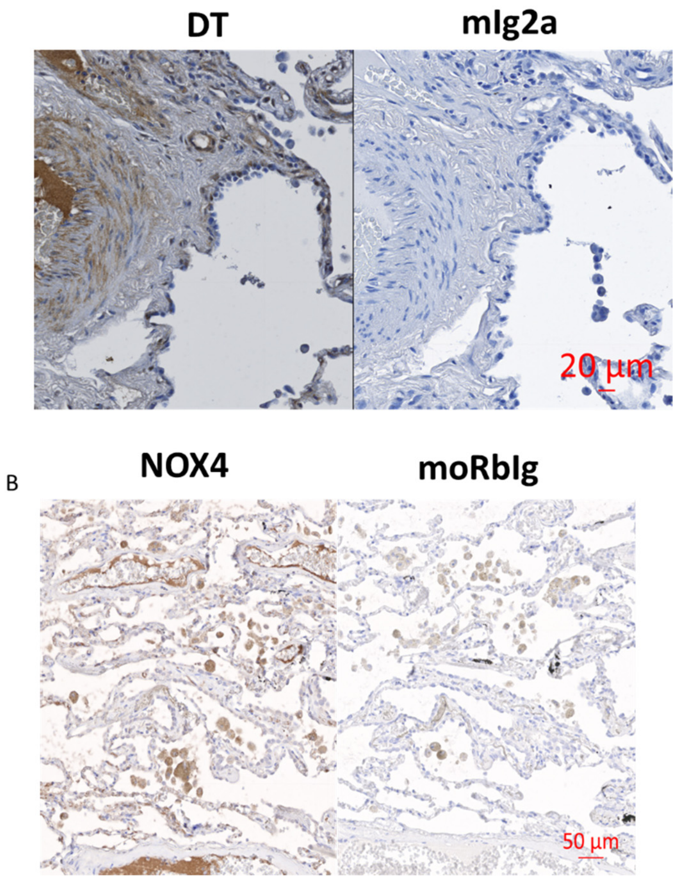

Figure S2. Representative images of DT (**A**) and NOX4 (**B**) staining in fibrotic regions of IPF/UIP samples and comparison were their respective isotype antibody controls.
